# Insight into the Salivary Gland Transcriptome of *Lygus lineolaris* (Palisot de Beauvois)

**DOI:** 10.1371/journal.pone.0147197

**Published:** 2016-01-20

**Authors:** Kurt C. Showmaker, Andrea Bednářová, Cathy Gresham, Chuan-Yu Hsu, Daniel G. Peterson, Natraj Krishnan

**Affiliations:** 1 Institute for Genomics, Biocomputing and Biotechnology, Mississippi State University, Mississippi State, Mississippi, 39762, United States of America; 2 Department of Biochemistry, Molecular Biology, Entomology and Plant Pathology, Mississippi State University, Mississippi State, Mississippi, 39762, United States of America; 3 Institute of Entomology, Biology Centre, Academy of Sciences, Branišovská 31, 370 05 České Budĕjovice, Czech Republic; 4 Department of Plant & Soil Sciences, Mississippi State University, Mississippi State, Mississippi, 39762, United States of America; USDA-ARS, UNITED STATES

## Abstract

The tarnished plant bug (TPB), *Lygus lineolaris* (Palisot de Beauvois) is a polyphagous, phytophagous insect that has emerged as a major pest of cotton, alfalfa, fruits, and vegetable crops in the eastern United States and Canada. Using its piercing-sucking mouthparts, TPB employs a “lacerate and flush” feeding strategy in which saliva injected into plant tissue degrades cell wall components and lyses cells whose contents are subsequently imbibed by the TPB. It is known that a major component of TPB saliva is the polygalacturonase enzymes that degrade the pectin in the cell walls. However, not much is known about the other components of the saliva of this important pest. In this study, we explored the salivary gland transcriptome of TPB using Illumina sequencing. After *in silico* conversion of RNA sequences into corresponding polypeptides, 25,767 putative proteins were discovered. Of these, 19,540 (78.83%) showed significant similarity to known proteins in the either the NCBI nr or Uniprot databases. Gene ontology (GO) terms were assigned to 7,512 proteins, and 791 proteins in the sialotranscriptome of TPB were found to collectively map to 107 Kyoto Encyclopedia of Genes and Genomes (KEGG) database pathways. A total of 3,653 Pfam domains were identified in 10,421 sialotranscriptome predicted proteins resulting in 12,814 Pfam annotations; some proteins had more than one Pfam domain. Functional annotation revealed a number of salivary gland proteins that potentially facilitate degradation of host plant tissues and mitigation of the host plant defense response. These transcripts/proteins and their potential roles in TPB establishment are described.

## Introduction

Insect saliva can perform numerous functions, though, one of its main roles is to initiate digestion [1, 2]. The salivary gland proteins of phytophagous insects include a complement of digestive proteins that can differ depending on feeding strategy. In phytophagous insects with chewing mouthparts, the saliva is used primarily to initiate digestion of the plant during the mastication process. On the other hand, in insects with piercing, sucking mouthparts, the saliva is injected into plant tissues where it solubilizes cellular and extracellular materials in preparation for eventual ingestion, through the insect’s stylet [3]. This mode of feeding has been described as “lacerate and flush”. Additionally, saliva of phytophagous insects can contain proteins and other molecules that can act as “effectors” to facilitate stylet penetration while suppressing host defense responses [4–9]. Thus, the insect saliva is the first metabolically active chemical substance that comes into direct contact with the plant and has a very important role in both food ingestion as well as insect-plant interactions [7, 10, 11].

The mirids (Order: Hemiptera, Sub-Order: Heteroptera, Family: Miridae) typically employ the “lacerate and flush” feeding strategy. The most well-known mirids are agricultural pests with a wide host range. Within the subfamily Mirinae are the lygus bugs (*Lygus* spp.), the species that cause the most economic damage to agronomically important plants. The tarnished plant bug (TPB) *Lygus lineolaris* (Palisot de Beauvois), is a highly polyphagous, phytophagous insect, with documented feeding on more than 300 plant species, collectively representing 36 plant families [12, 13]. Plant symptoms resulting from TPB feeding include organ abscission, deformation of developing fruits, necrosis at the feeding site, seeds with aborted embryos, and reduced vegetative growth [14].

The TPB is notorious for causing numerous physiological effects in cotton including: shortened internode length, apical termination, loss of apical dominance leading to the development of many secondary terminals (*crazy cotton*), anther necrosis, square abscission, yellowish staining of the apex developing floral bud (*dirty square*), as well as the yellowish staining of the anthers (*dirty flower*), and complete cellular dissolution of the floral bud [15–18]. Several of these effects are due to TPB’s preferential feeding on the cotton floral structure as it develops [16–18]. With the broad-scale adoption of genetically modified *Bacillus thuringiensis* (Bt) cotton and the eradication of the boll weevil, insecticide applications to control TPB have increased [16]. Moreover, TPB populations have been shown to be increasingly resistant to some insecticide classes commonly used for their control including cyclodienes, organophosphates, pyrethroids, and neonicotinoids [19–23]. Insecticide resistance and the ability of TPB to enter diapause, which permits insects to overwinter have made TPB a difficult pest to control [24]. Economic thresholds based on TPB counts from sweeps and drop cloths as well as counts of *dirty squares* have been established to assist growers in determining whether TPB countermeasures are economically advisable [25–27]. Of interest, the *dirty square* symptom that develops into the *dirty flower*, appears to be the manifestation of molecular catabolism caused by TPB feeding. This symptom is also used for resistance screening in cotton cultivar and germplasm breeding programs since phenotypic evaluations of cotton plants have suggested that resistance to *dirty square* (i.e. resistance to TPB) is under genetic control [28–32].

The role of pectin degrading enzymes (polygalacturonases) in the saliva of lygus bugs was recognized almost four decades ago [14], however conclusive evidence for the specific role of polygalacturonases in causing lygus-like damage to plants came much later from studies of the western tarnished plant bug (WTPB) *L*. *hesperus* [33, 34]. The study of de la Paz Celorio-Mancera et al. 2008 [35] suggested the presence of four polygalacturonases (*Lhpg1*, *Lhpg2*, *Lhpg3* and *Lhpg4*) in salivary gland extracts of the WTPB. Whereas in TPB three polygalacturonase encoding genes (*Llpg1*, *Llpg2* and *Llpg3*) were described [36]. RNAi mediated knockdown of *Llpg1* in TPB displayed no obvious phenotype, with no detrimental effects on longevity [37].

Many of the earlier salivary gland transcriptome analysis conducted in the last decade mainly focused on blood feeding insects such as *Ixodes scapularis* [38, 39], *Anopheles gambiae* [40–43], *Dermacentor andersoni* [44], *Triatome brasiliensis* [45] and *Amblyomma variegatum* [46] among others. However, some of the more recent studies on salivary gland transcriptomes have focused on phytophagous insects such as the potato leaf hopper, *Empoasca fabae* [5], the green rice leaf hopper, *Nephotettix cincticeps* [47] and the western flower thrips, *Frankliniella occidentalis* [48]. For the mirid bugs, a study utilizing whole body transcriptome assembly of *Lygus hesperus* [49] and an EST library of *Apolygus lucorum* [50] was recently conducted. However, to our knowledge there is a lack of detailed information on the salivary gland transcriptome of TPB and neither does a complete genome sequence exist despite its economic importance.

A detailed proteomic analysis of the salivary proteins of the WTPB was recently conducted, which led to the identification of laccase, glucose dehydrogenase and xanthine dehydrogenase, in addition to the normal complement of PGs, α- amylases, and proteases [51]. The authors proposed that these newly identified enzymes could target plant-defense compounds.

Previous genetic studies of the TPB have used microsatellite markers to explore the genetic diversity of TPB populations from different geographical regions [52, 53]. To date, studies of transcripts have focused on RNAs isolated from the whole body or the gut [54–57]. Recently, a transcriptome was assembled for WTPB as a means to investigate the mechanisms of thermal tolerance [49].

In order to devise suitable genetic strategies for the effective control of TPB, it is important to have an in-depth knowledge of gene expression in salivary glands. While analysis of saliva has yielded considerable information on saliva contents, whole salivary gland transcriptomics should help reveal gene pathways involved in controlling saliva composition, production, and secretion. Such information, in turn, may help in the development of strategies that target specific TPB gene pathways involved in TPB-plant interactions. In this investigation, we present the salivary gland transcriptome (sialotranscriptome) of TPB. Additionally, we discuss gene expression patterns and metabolic pathways that could be targeted in reducing TPB-based damage.

## Results and Discussion

### RNA extraction, Illumina library preparation and sequencing

**Fig 1:**
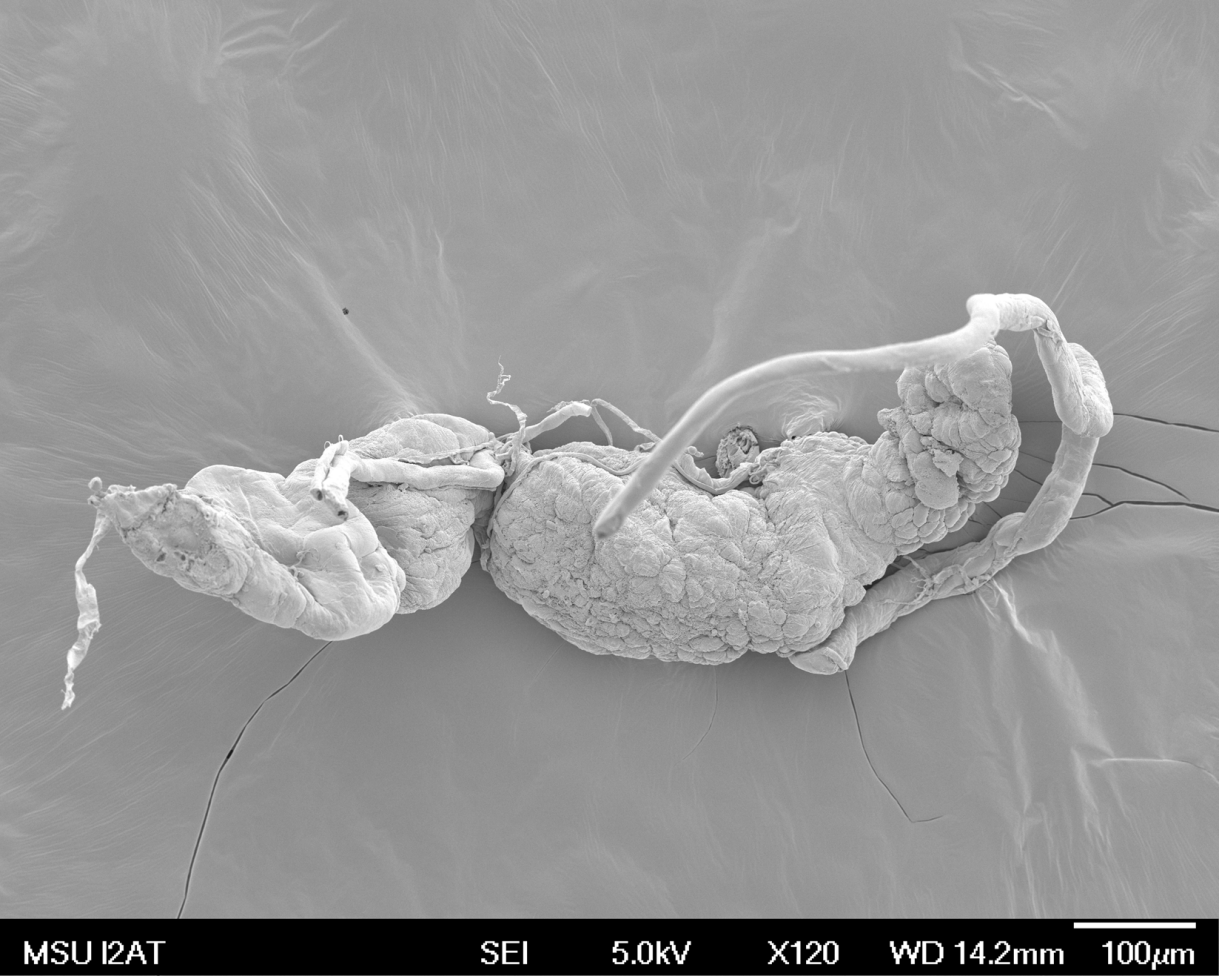
Scanning electron micrograph of excised salivary glands of the tarnished plant bug *Lygus lineolaris*.

RNA extractions from the 3 pools of 25 gland pairs (Fig 1) yielded an average of 6 μg of total RNA. Non-denatured agarose gel electrophoresis and Agilent Bioanalyzer 2100 analysis (with Agilent RNA 6000 Nano kit) showed one predominant rRNA peak near 0.9 kb, characteristic of 28S rRNA cleavage and co-migration of the alpha 28 and beta 28 subunits with the 18S rRNA, a phenomena commonly observed in insect RNA isolations (S1 Fig) [58]. Illumina double-stranded library preparation yielded 1.4, 1.32, and 1.34 μg of library products for the respective pools with fragment sizes of 213, 207, and 212 bp, respectively. After sequencing, a total of 18,389,715 read pairs (paired ends) passed minimum quality requirements. Collectively, these paired end reads represent a total of 7,355,886,000 base pairs of sequence data.

### Short read transcriptome assembly

Read trimming with Trimmomatic [59] resulted in 24,744,346 (67.3%) reads meeting quality requirements. Of these, 19,368,620 (52.7%) reads remained paired and 5,375,726 (14.7%) were placed into unpaired single ends. Trinity [60, 61] transcriptome assembly of trimmed paired and unpaired reads resulted in the assembly of 77,171 transcripts. Curation of the assembled transcriptome by the methods of Yang and Smith (2013) [62], yielded 19,884 transcripts with BLASTX alignments to 403,496 different proteins in the Pterygota (winged insect) database downloaded from the UniProtKB [63]. Alignments allowed detection and subsequent trimming of 61 chimeric transcripts with the python scripts described by Yang and Smith 2013 [62]. Post assembly curation resulted in the final transcriptome assembly described in this study. This transcriptome called TPB_SG1, is composed of 62,559 transcripts with an N50 of 817 bp and a total size of 36.12 Mb (Table 1).

**Table 1:** Summary statistics of the sialotranscriptome of *Lygus lineolaris*.

Total number of raw reads	36,779,430
Total number of trimmed reads (clean)	24,744,346
Total base pairs in assembly	36.12 x 10^6^
Read length	200 bp (paired end)
Total number of contigs	62,559
Mean length of contigs	577
N50 contig length	817
Minimum length	201
Maximum length	13,408

### Protein prediction and annotation

**Fig 2:**
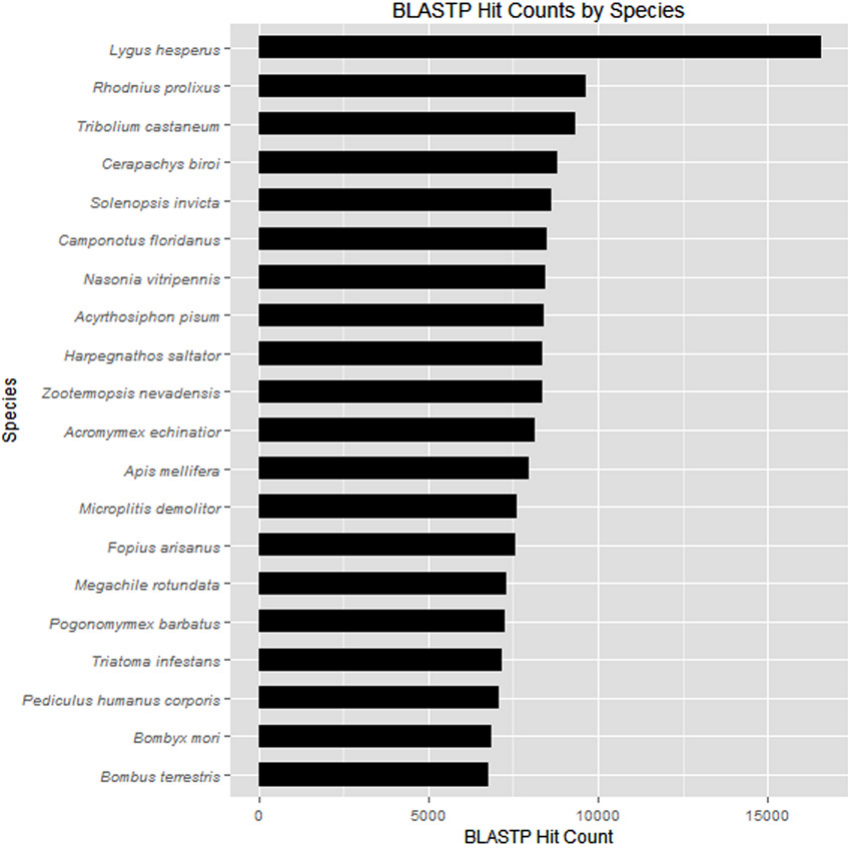
Top 20 species exhibiting alignments (E-value ≤1.00E-05) to the *L*. *lineolaris* sialotranscriptome.

A total of 25,767 proteins were predicted (Table 2) on the sense strand of the transcripts with Transdecoder [61]. The predicted proteins had a total of 883,061 and 858,136 qualifying alignments to the NCBI nr and Uniprot-KB databases, respectively. The species showing the most BLASTP alignments to the TPB transcriptome were *Lygus hesperus* (n = 16,559), *Rhodnius prolixus* (n = 9,632), *Tribolium castaneum* (n = 9,335), and *Cerapachys biroi* (n = 8,824) (Fig 2) (S1 Table). GO association mapping from the BLASTP alignments yielded 11,090 GO terms to 1,097 predicted proteins. Interproscan identified 5,998 unique InterPro IDs for 11,694 predicted proteins and 20,332 GO terms to 7,512 predicted proteins. Of the 803 predicted proteins with a predicted signal peptide, 663 proteins were classified as being secreted since they did not contain transmembrane helices.

**Table 2:** *Lygus lineolaris* TPB_SG1 assembly predicted protein annotation summary.

**Predicted Proteins**	25,767
**Protein Completeness**	
Complete	7,069
5' partial	5,512
3' partial	3,591
Internal	9,595
**Proteins with BlastP Alignments**	
NR	15,600
Uniprot	19,526
Either NR or Uniprot	19,540
Neither NR or Uniprot	6,227
**Pfam Annotations**	12,851
**KEGG Annotations**	791
**GO Annotations**	31,240
**GO terms assigned by BlastP ID mapping**	11,090
**GO terms assigned by InterProScan**	20,150
**GO Biological Process terms**	13,405
**GO Molecular Function terms**	12,019
**GO Cellular Component terms**	5,816
**Proteins with SignalP**	803
**Proteins with SignalP w/o TMHMM**	663

### Comparative analysis

**Fig 3:**
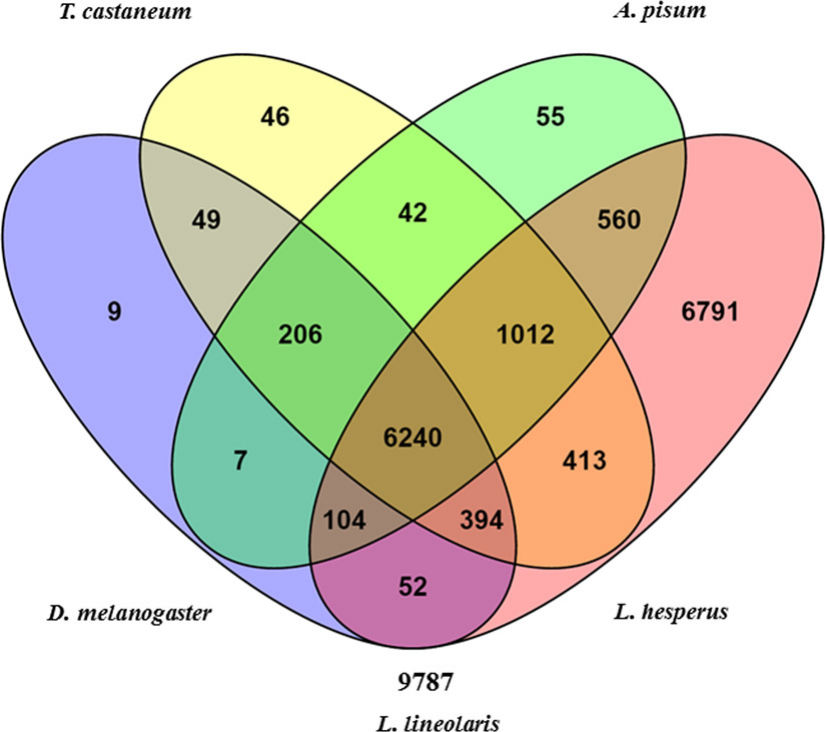
Venn diagram showing distribution of 15,980 *L*. *lineolaris* salivary gland proteins with BLASTP alignments to *Acyrthosiphon pisum*, *Drosophila melanogaster*, *Tribolium castaneum*, and *Lygus hesperus*.

The predicted protein sequences derived from the salivary gland transcriptome of TPB were compared to protein sequences of WTPB as well as model insect species such as the fruit fly *Drosophila melanogaster* (Diptera), the confused flour beetle *Tribolium castaneum* (Coleoptera) and the pea aphid *Acyrthosiphon pisum* (Hemiptera) (Fig 3) [64–66]. Approximately 31.9% (8,226 BLASTP hits) of the 25,767 protein sequences of TPB exhibited sequence similarity with proteins from *A*. *pisum*, 32.6% (8, 402 BLAST hits) showed sequence similarity to *T*. *castaneum*, and 27.4% (7,061 BLASTP hits) shared significant similarity to proteins from *D*. *melanogaster* (Fig 3). Interestingly, within the blast results, *D*. *melanogaster* core RNA interference processing proteins Dicer2 Argonaute2 and loquacious were identified. Also, while TPB exhibited 6,446 sequences with high similarity to sequences in all three non-Hemipteran insects, it shared 15,566 BLASTP hits with WTPB (60.4%). The TPB_SG1 transcriptome had a CEGMA completeness of 90.32% for complete proteins and 94.35% when including partial proteins, which is comparable to other insects (Table 3).

**Table 3:** CEGMA completeness report summary.

	Number of complete proteins	% Complete	Number of partial proteins	% partial
**TSA assemblies**				
*Lygus hesperus*	239	96.37	242	97.58
*Lygus lineolaris*	224	90.32	234	94.35
**Genome assemblies**				
*Acyrthosiphon pisum*	230	92.74	243	97.98
*Drosophila melanogaster*	241	97.18	245	98.79
*Tribolium castaneum*	220	88.71	293	96.37

### Gene ontology

**Fig 4:**
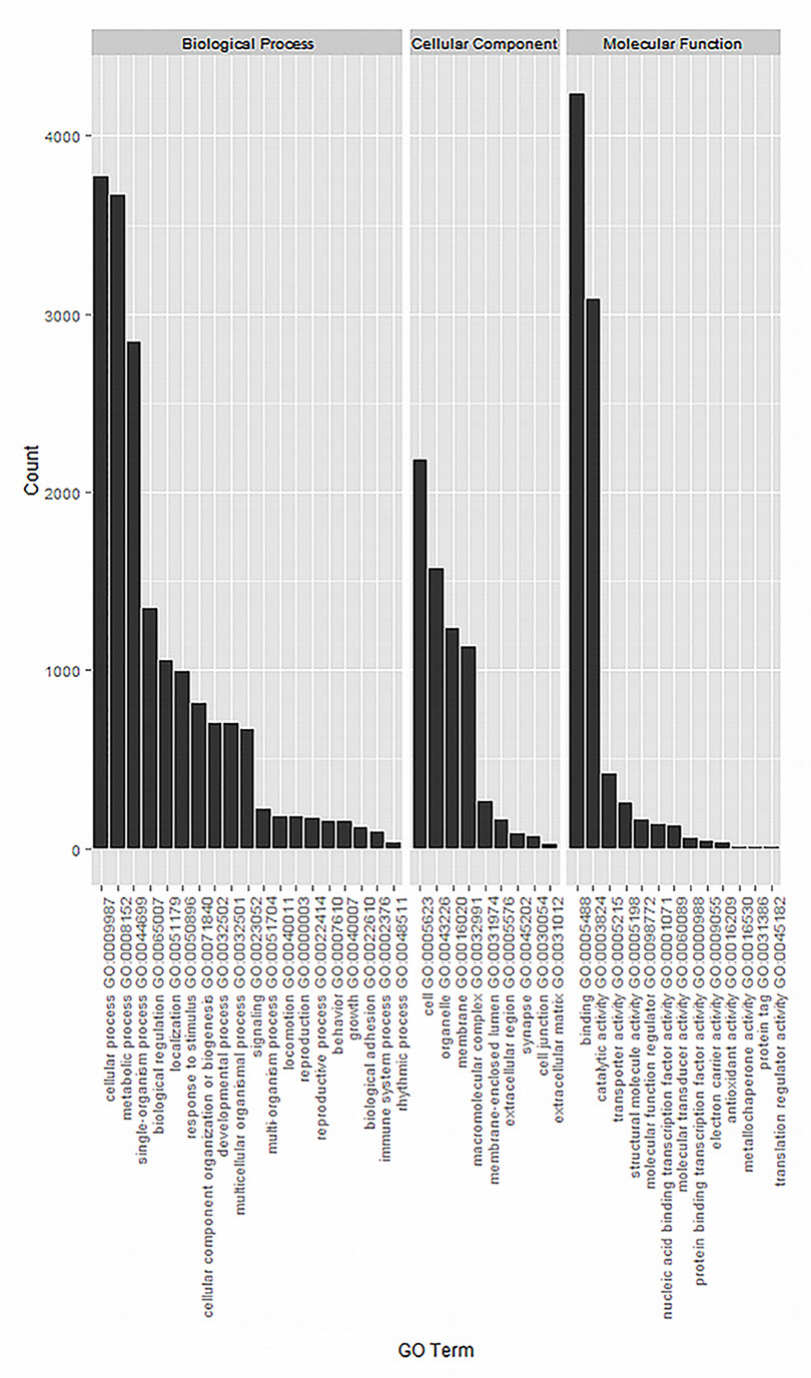
Classification of *L*. *lineolaris* salivary gland transcriptome based on predicted Gene ontology (GO) terms. (**A**) Biological Processes (**B**) Molecular Function (**C**) Cellular Component.

GO terms arising from both the BLASTP and InterProScan (ISA and IEA evidence codes, respectively) were combined for further analysis (S2 Table). The set of combined GO terms were subsequently summarized to GO term level 2 (Fig 4). In the category of biological processes there were 17,855 terms with the three most common categories assigned being cellular processes (3,773), metabolic processes (3,668) and single organism processes (2,842) (Fig 4). In the molecular function category (8,543 terms), the top three categories assigned were binding (4,237), catalytic activity (3,087) and transporter activity (415) (Fig 4). Finally, in the cellular component category (6,703 terms), the three predominant categories assigned are cell (2,183), organelle (1,566) and membrane (1,234). These most common assigned categories are consistent with other sialotranscriptomes among a wide range of phytophagous insects including; *Bemisia tabaci*, *Empoasca fabae*, *Frankliniella occidentalis*, *Helicoverpa armigera*, and *Nilaparvata lugens* [5, 48, 67, 68]. GO term enrichment for the predicted secreted proteins identified molecular function terms such as hydrolase activity (GO:0004553), polygalacturonase activity (GO:0004650), peptidase activity (GO:0008233), serine-type endopeptidase activity (GO:0004252) and catalytic activity (GO:0003824) to be overrepresented in the proteins predicted to be secreted (S3 Table). This high representation of GO molecular functions terms relating to proteolysis and degradation emphasizes the role of saliva in digestion, while providing further evidence of the physiological role of the saliva secreted by the SGs in the TPB herbivory.

### KEGG pathway

The Kyoto Encyclopedia of Genes and Genomes database was used to identify potential pathways represented in the transcriptome [69, 70]. A total of 791 proteins in the salivary gland transcriptome of TPB were mapped to a total of 107 KEGG pathways (S4 Table), the top 20 of which are depicted in Table 4. The majority of the salivary gland transcript sequences mapped to metabolic pathways including purine metabolism (239 predicted protein sequences with 34 enzymes), pyrimidine metabolism (229 predicted protein sequences with 26 enzymes) and aminoacyl-tRNA biosynthesis (77 predicted protein sequences with 22 enzymes). The presence of the latter indicates a high level of protein synthesis activity occurring in the SGs of the TPB. Consistent with the lacerate and flush feeding strategy, several of the enzymes found in predicted KEGG pathways are involved in sugar metabolism including: amino sugar and nucleotide sugar metabolism (17 enzymes with 41 predicted protein sequences) and glycolysis/gluconeogenesis (18 enzymes with 36 predicted protein sequences). Interestingly, there were also 12 enzymes with 48 predicted protein sequences which were involved with glutathione metabolism. This is consistent with the antioxidant Molecular Function roles assigned to 31 transcripts (Fig 4). It is expected that this high level of antioxidant activity would be necessary to quell the herbivory induced plant defenses which often involve reactive oxygen species [71–73].

**Table 4:** Top 20 predicted KEGG pathways in the *L*. *lineolaris* sialotranscriptome.

KEGG pathway	Number of enzymes	Number of predicted proteins
Purine metabolism	34	239
Pyrimidine metabolism	26	229
Aminoacyl-tRNA biosynthesis	22	77
Glycolysis / Gluconeogenesis	18	36
Amino sugar and nucleotide sugar metabolism	17	41
Citrate cycle (TCA cycle)	15	28
Pentose phosphate pathway	15	27
Pyruvate metabolism	15	22
Carbon fixation pathways in prokaryotes	15	23
Glycerophospholipid metabolism	14	24
Cysteine and methionine metabolism	13	23
Methane metabolism	13	21
Alanine, aspartate and glutamate metabolism	12	15
Arginine and proline metabolism	12	17
Glutathione metabolism	12	48
Terpenoid backbone biosynthesis	11	20
Drug metabolism—other enzymes	10	18
Glycine, serine and threonine metabolism	10	18
Porphyrin and chlorophyll metabolism	10	12
Carbon fixation in photosynthetic organisms	10	21

### Protein domains

We identified 3,653 Pfam domains in 10,421 salivary gland transcriptome-predicted proteins resulting in 12,814 Pfam annotations (S5 Table). Among the top 20 Pfam domains were reverse transcriptases (RNA-dependent DNA polymerases) (281 proteins) indicative of retrotransposons of plant (Ty-1 copia or Ty-3 gypsy) or insect origin and/or retrovirus integrations (e.g. LyLV-1) in the TPB genome [74]. Reverse transcriptases allow retrotransposons to copy themselves to RNA and back to DNA that may integrate back to the genome. Zinc-finger double domains which help in protein dimerization (194 proteins) and are also involved in various cellular processes were noted in the salivary gland transcriptome (Table 5). Protein domains associated with sugar and other transporters were also identified (74 proteins). Fifty-seven TPB_SG1 transcripts associated with protein domains related to cytochrome P450 and 31 transcripts associated with protein domains of carboxylesterase were identified (S5 Table); unlike the sialotranscriptome of the potato leaf hopper [5], cytochrome P450 and carboxylesterase domains were not in the top 20 domain classes recognized by TPB_SG1. One-hundred-and-fifty TPB_SG1 transcripts are associated with the large and rapidly expanding protein family known as the WD-domain G-beta repeats [75]. The WD-repeat proteins participate in biological processes such as signal transduction, transcription regulation, and apoptosis, although the specific function of the WD-repeat domain is unclear [75]. More knowledge of WD-repeat proteins is critical to understanding many biological processes including those that take place in the sialotranscriptome of TPB.

**Table 5:** Top Pfam domains identified in *L*. *lineolaris* sialotranscriptome predicted proteins.

PFAM ID	PFAM ID Description	Number of proteins

PF00078	Reverse transcriptase (RNA-dependent DNA polymerase)	281
PF13465	Zinc-finger double domain	194
PF00665	Integrase core domain	187
PF00096	Zinc finger, C2H2 type	166
PF00069	Protein kinase domain	163
PF00400	WD domain, G-beta repeat	150
PF13894	C2H2-type zinc finger	93
PF07727	Reverse transcriptase (RNA-dependent DNA polymerase)	91
PF00076	RNA recognition motif. (a.k.a. RRM, RBD, or RNP domain)	87
PF12796	Ankyrin repeats (3 copies)	79
PF00083	Sugar (and other) transporter	74
PF00651	BTB/POZ domain	72
PF00271	Helicase conserved C-terminal domain	71
PF07679	Immunoglobulin I-set domain	68
PF00089	Trypsin	62

### Proteins of interest

The TPB sialotranscriptome was screened for several known salivary proteins found in phytophagous insects (S6 Table). Sequence comparisons revealed 36 serine protease snake-like proteins, 31 ATP-dependent zinc metalloproteases, 28 polygalacturonases, 16 cathepsins, and 16 esterase FE-4-like proteins (Table 6). The salivary protein encoding transcripts/ proteins were categorized as those involved in general digestion, sugar metabolism, cell wall digestion, immunity-related responses, detoxification, suppression of plant defense responses, and other phytophagy processes (specific functions unknown). Interestingly, pectin lyases and endoglucanases were not present in the sialotranscriptome of TPB; such proteins were observed in the sialotranscriptome of *E*. *fabae*, the potato leaf hopper [5]. However, we did record other glycan degrading enzymes that would possibly target the hemicellulose though they were not represented in the top 20 salivary proteins / enzymes (S4 and S5 Tables). The high representation of serine protease snake-like proteins (36 proteins) in the TPB_SG1 dataset was a bit surprising as these putative immune-response proteins were reported earlier from an immunogenomics study in the squash bug, *Anasa tristis* (De Geer) [76]. However, serine protease snake-like proteins were represented in the sialotranscriptome of the rice brown plant hopper, *N*. *lugens* [68]. In addition to transcripts/proteins involved in general digestive processes and sugar metabolism, genes involved in degradation of plant cell wall components and those involved in detoxification and inhibition of plant defense responses were well represented in the TPB sialotranscriptome (Table 6).

**Table 6:** Genes of interest identified in the *L*. *lineolaris* sialotranscriptome.

Functional category of genes	Candidate genes	Proteins	Number of TPB SG1 BlastP Hits
**General digestion**	cathepsin-L, partial	ABF18889.1	16
	esterase FE4-like	XP_001943214.2	16
	cathepsin-L-like cysteine proteinase 2	ABF18890.1	12
	probable chitinase 3 isoform X1	XP_001943038.2	12
	short/branched chain specific acyl-CoA dehydrogenase, mitochondrial	XP_001947176.2	8
	lipase 3-like isoform X2	XP_003246825.1	4
	granzyme-like protein 1	XP_003247717.1	3
	carboxypeptidase D-like	XP_001952348.1	2
**Sugar metabolism**	similar to Lactase-phlorizin hydrolase precursor (Lactase-glycosylceramidase)	XP_001945606.1	12
	sucrase	ABB55878.1	7
	lysosomal alpha-glucosidase-like	XP_001952631.1	3
	Alpha-amylase_C	BAH72207.1	1
**Extra-oral digestion of cell wall components**	polygalacturonase	ACC44799.1 ACC44798.1 ABD63920.1 ABD63922.1 ACZ28127.1	28
	uncharacterized family 31 glucosidase KIAA1161-like	XP_001952674.2	5
**Immune related**	serine protease snake-like	XP_003247331.1	36
**Detoxification and inhibition of plant defenses**	ATP-dependent zinc metalloprotease YME1 homolog isoform X1	XP_001946697.2	31
	glucose oxidase	NP_001011574.1	11
	Xanthine dehydrogenase	P08793.1	4
	regucalcin-like	NP_001155519.1	3
	laccase-7-like & laccase 2	XP_001946224.1 BAJ83488.1	3
**Unknown function**	unknown [*Lygus lineolaris*]	ABQ18257.1	4
	unknown [*Lygus lineolaris*]	ABQ18254.1	3
	apyrase-like, partial	XP_001947326.3	1

**Fig 5:**
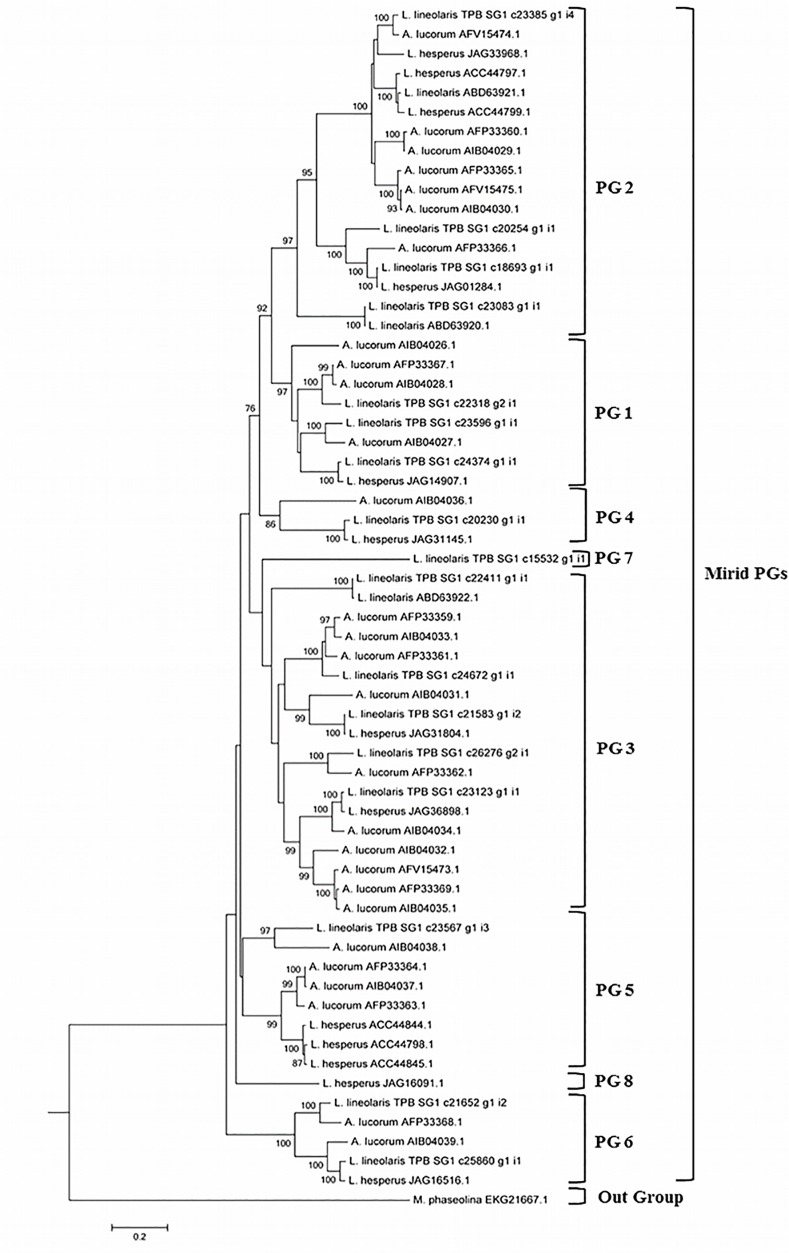
Phylogenetic relationships of polygalacturonase transcripts of *L*. *lineolaris* with polygalacturonases from other mirid bugs.

Twenty eight polygalacturonase proteins were identified in the TPB sialotranscriptome, of which 17 were predicted full-length protein sequences. A neighbor-joining tree using Poisson-correction distances was constructed from a multiple sequence alignment using the full length polygalacturonase proteins from mirid bugs including TPB, WTPB, and *Apolygus lucorum*, where the *Macrophomina phaseolina* glycoside hydrolase (GH) domain was used as an out group. The mirid bug polygalacturonases in this study were divided into 8 clusters (polygalacturonase 1–8) of which 7 clusters contained representatives from the TPB sialotranscriptome (Fig 5). The previously described TPB polygalacturonase proteins ABD63920 and ACZ28127 clustered into the polygalacturonase 2 cluster that contained four TPB salivary gland transcriptome polygalacturonase protein sequences. The other previously described TPB polygalacturonase protein ABD63922, clustered into the polygalacturonase 3 cluster, where it paired with a TPB_SG1 protein. Polygalacturonase 8 from the WTPB formed a cluster by itself.

The plant cell wall is a resilient and structurally heterogeneous barrier composed of complex polysaccharides and diverse proteins [77]. The main components of the primary cell walls are members of two polysaccharide networks, consisting of cellulose and hemicellulose embedded in a pectin polysaccharide matrix [78]. To breach this complex barrier, herbivorous insects have evolved a remarkable array of polysaccharide degrading enzymes, including exo- and endo-polygalacturonases, pectin methyl-esterases, pectin lyases and pectate lyases, acetyl esterases, xylanases, and a variety of endoglucanases that cleave cellulose, xyloglucan, and other glucans [79–81]. The term *cellulases* is used to describe cellulolytic enzymes such as endogluconases, exogluconases, and β-glucosidases. In addition to cellulases, degradation of plant cell walls requires pectinases and hemicellulases. These are all grouped into the glycoside hydrolase (GH) family according to their amino acid sequence similarities and their folding patterns based on the Carbohydrate-Active enZymes Database [82]. Cellulases in the GH9 family are found in most insect orders while polygalacturonases of the GH28 family have a much more restricted distribution in insects [83]. Homogalacturonan polymers are the main components of pectin in primary cell walls, and the polygalacturonases identified in this study presumably cleave the 1,4-linkages of the homogalacturonan α-D-galacturonic acid [83]. A phylogenetic analysis has revealed that the polygalacturonases of the mirid bugs cluster with the ascomycete Pezizomycotina + Saccharomycotina clade with yeasts (Saccharomycetacease) as an outgroup [79]. The GH28 family of glycosyl hydrolases includes proteins with different enzymatic specificities including polygalacturonases, rhamnogalacturonases, and xylogalacturonases, but based on phylogenetic analyses and conservation of amino acid residues it appears that this entire gene family had a single origin in the tree of life [83, 84]. It has been demonstrated however that some conserved amino acid sequences enable a distinction between polygalacturonasess and other pectolytic enzymes of the GH28 family [85].

As previously suggested [79], it is possible that polygalacturonase genes in the mirids were obtained by horizontal gene transfer (HGT) as all mirid polygalacturonases have characteristic amino acids conserved in microbial and plant polygalacturonases. The large repertoire of polygalacturonase transcripts in the TPB sialotranscriptome raises the interesting possibility that some of these may actually be translated into catalytically inactive proteins that can act as “decoy” targets for plant derived polygalacturonase inhibitory proteinases suggested earlier [86]. However, this hypotheses remains to be tested. Current work in our laboratory is underway to test if all the PG transcripts eventually translate to functional proteins or if some of them serve as “decoys”.

The second most common sequences in the TPB sialotranscriptome were ATP-dependent zinc metalloproteases. These enzymes have been implicated in mitochondrial protein metabolism and protection of mitochondria from the accumulation of oxidatively damaged membrane proteins [87]. The presence of ATP-dependent zinc metalloproteases is consistent with the fact that herbivory would induce the formation of reactive oxygen species (ROS) at localized areas in the plant tissue, and such ROS could cause damage to the insect if not neutralized [72].

Eleven protein sequences of glucose oxidase (GOX EC 1.1.3.4) were found in the TPB sialotranscriptome. GOX is the first insect salivary enzyme that has been shown to suppress wound-inducible herbivore defenses of plants [88]. Salivary GOX secreted by *Helicoverpa zea* was demonstrated to inhibit wound induced nicotine production in *Nicotiana tabacum* as well as delayed–induced defenses in the tomato plant *Solanum lycopersicum* [88–90]. Salivary GOX is found in many caterpillar species and also in aphids [6, 91, 92]

Signature Pfam domains (PF00199, PF10417) for ROS scavenger enzymes such as catalase (1.11.1.6) and peroxiredoxin (1.11.1.15) were identified in the salivary gland transcriptome. Insect feeding has been shown to increase peroxidase activity in the cotton leaves and sap [93]. The cotton transcript encoding catalase has previously been shown to be down regulated in response to feeding by both a sap-feeder, *Aphis gossypii* and a chewing insect, *Bemisia tabacci* [94]. Thus, we hypothesize that through salivary GOX and ROS scavengers, TPB may be limiting ROS production and suppressing ROS-triggered defensive responses in the host plants. Such a strategy would help the TPB successfully establish itself by suppressing host-plant induced defenses during feeding.

Xanthine dehydrogenase (XDH) has been reported in insects and has been most well characterized in *D*. *melanogaster* and the silkworm *Bombyx mori* [95–99]. In contrast to XDH in vertebrates, insect XDH does not appear to be converted into xanthine oxidase. Insect XDH functions in the catabolism of purines and detoxification of dietary purines which can be converted into the powerful antioxidant uric acid in the presence of NAD+. Thus, XDH and uric acid are secreted on the mucosal surface of the salivary gland thereby expanding their function extracellularly and into the saliva [100]. Interestingly, three regucalcin-like transcripts were also found in the sialotranscriptome of the TPB. Regucalcin is a Ca^2+^ binding protein that has been also reported in the saliva of *A*. *pisum*, *B*. *tabaci* and *F*. *occidentalis* [48, 67, 101, 102].It is hypothesized that regucalcin might have a role in regulation of calcium mediated signaling or in inhibiting calcium-mediated defenses in plant cells in response to herbivory [103]. Several genes with unknown functions were also found such as apyrase-like (1 protein). It is unclear what function the apyrase-like gene would perform in the saliva of a phytophagous insect. In blood sucking insects, salivary apyrases have been reported to play a role in inhibiting ADP and inhibition of ADP-induced platelet aggregation [2, 104]. When the TPB apyrase-like protein was aligned to the NCBI nr database it showed the highest similarity to an apyrase-like protein present in the bean bug, *Riptortus pedestris*, suggesting that the function of this protein is likely shared among other phytophagous insects. Apyrases are also been known to be ATP degrading enzymes and they can modulate the level of extracellular ATP (eATP) which can impact pathogen defense response in plants [105–107]. For example, apyrase in TPB saliva may degrade eATP in host plants that may impact cell viability and death as well as compromise basal resistance to viral and bacterial infection.

Interestingly some endonucleases were detected but again these were not represented to the extent that they figured in the top 20 proteins of interest. It has been demonstrated earlier that the saliva of TPB digests double stranded ribonucleic acids [108]. We presume that the endonucleases we detected (showing homology to dicer2 and argonaute2 which are known nucleases) could possibly function in degrading the double stranded ribonucleic acids. This would pose a problem for delivery of dsRNA targeted against any major gene in TPB since they would be rapidly degraded by saliva and have very limited persistency in the digestive tract.

## Conclusions

Our transcriptomic analysis of the salivary glands of TPB provides biological insights into the means by which this mirid bug establishes a feeding site on a host plant. Catalytic enzymes associated with digestion were highly represented in the TPB sialotranscriptome, which is consistent with the extra-oral digestion and solubilization of nutrients from the plant tissue. In addition to salivary proteins and enzymes involved in general metabolism, we also show the presence of several immune related transcripts with possible involvement in counteracting plant defense responses. Phylogenetic evidence revealed the complement of TPB polygalacturonase enzymes is similar to that of other mirid bugs such as *Apolygus lucorum* and the WTPB, and constitutes a large expanded family of polygalacturonases. While we have validated some of the transcripts given in this assembly (mainly the polygalacturonases), our future work is focused on elucidating the role of GOX and regucalcin in this important pest insect (data not shown) since these likely target host-plant defenses. The TPB sialotranscriptome is thus a valuable resource for further research on TPB and its interaction with host plants.

## Materials and Methods

### Insect collection and rearing

*Lygus lineolaris* (tarnished plant bugs or TPBs) used in this research were obtained from a long term colony housed at the Mississippi State University Insect Rearing Center. TPBs were reared in 8.3 L plastic containers (Rubbermaid Servin' Saver®) filled with shredded paper and equipped with self-sealing lids modified by removing the center of the lid. A fine mesh screen was placed on the containers and held down with the remaining portion of the self-sealing lid. Screen covers and shredded paper were replaced weekly. Insect colonies were kept in a rearing chamber maintained at 26.7 ± 2°C at an approximate relative humidity of 65 ± 5% with a 16:8 light: dark cycle. The insects were fed an oligidic artificial diet similar to that of Cohen (2000) presented on a 5 x 5 cm Parafilm (Beemis Company Inc., Neenah, WI), and placed on top of the screens [109]. The insects fed by puncturing the Parafilm. Food was replaced three times per week. Mixed sex adults were separated from the colony as soon as they eclosed and utilized for the experiments within 2–3 days

### RNA extraction, Illumina library preparation and sequencing

Three different Illumina libraries were generated. For each library, salivary gland pairs from 25 individuals were dissected on dry ice, total RNA was extracted using an RNeasy Mini Kit (CAT# 74104, Qiagen, Valencia, CA, USA), and RNA was treated with 20 units of Takara Recombinant DNase I (CAT# 2270A, Clontech Laboratories Inc., Mountain View, CA, USA). RNA integrity was assessed using agarose gel electrophoresis and the Agilent Bioanalyzer 2100 using Agilent RNA 6000 Nano kit (CAT# 5067–1511, Agilent Technologies, Palo Alto, CA, USA). Illumina strand specific libraries were prepared with the Illumina Stranded mRNA library construction kit (CAT# RS-122-2101, Illumina, San Diego, CA, USA) according to the manufacture’s protocol using 3 μg of total RNA. Molar concentrations of resulting DNA libraries were determined using fragment sizes determined via an Agilent Bioanalyzer 2100 with an Agilent DNA 1000 kit (CAT# 5067–1504, Agilent Technologies, Palo Alto, CA, USA). DNA concentrations were determined using Qubit fluorometer with the Qubit dsDNA HS assay kit (CAT# Q32851, Life Technologies, Grand Island, NY, USA). The three libraries were pooled at equal concentration and volume for 15 pM cluster generation followed by paired end (PE) 200 bp (2 X 200) sequencing on the Illumina MiSeq with the Illumina MiSeq Reagent kit v3 (CAT# MS-102-3003, Illumina, San Diego, CA, USA). All raw read data was deposited in the National Center for Biotechnology Information (NCBI) under BioProject PRJNA280549 and BioSample SAMN03464333 with Sequence Read Archive (SRA) [110] accession numbers; SRR1956749, SRR1956751, and SRR1956752.

### Short read transcriptome assembly

Raw demultiplexed reads were trimmed with Trimmomatic-PE software using parameters “ILLUMINACLIP: TruSeq3-PE.fa:2:30:10 LEADING: 28 TRAILING: 28 SLIDINGWINDOW: 4: 28 MINLEN: 140” [59]. All trimmed paired and unpaired reads were assembled together in a single assembly with Trinity release r20140717 using the strand specific flag and minimum contig length of 200 bp option [60]. Subsequent assembly curation was performed to remove redundant transcripts and trim miss-assembled chimeras as described by Yang and Smith (2013) [62]. Briefly, RNA-Seq by Expectation Maximization (RSEM, version 1.2.15) values calculated for Bowtie2 (v 2.2.5) mapping of trimmed paired reads were determined for each transcript using the perl script align_and_estimate_abundance.pl provided in the Trinity release [61, 111, 112]. Subsequently, the highest expressed isoform for each Trinity component (gene) was chosen as a representative of all the isoforms within the subcomponent assignment. The resulting highest RSEM isoform transcripts were aligned with BLASTX (NCBI BLAST version 2.2.29) to all the Insecta sub-class Pterygota (Lang 1888) (winged insects) 1,097,562 protein sequences available in UniProtKB (downloaded 11/14/14) using parameters previously described in Yang and Smith (2013) [62]. The BLASTX alignments with the Pterygota database was used in chimera identification and trimming using the python scripts described in Yang and Smith (2013) [62]. Transcripts containing a BLASTN hit to Illumina adapter sequences in the NCBI Univec database using VecScreen specific parameters were removed from the assembly. The resulting transcripts that met repository requirements were deposited as a Transcriptome Shotgun Assembly (TSA) in DDBJ/EMBL/GenBank under the accession GDAW00000000. The version described in this paper is the first version, GDAW01000000 (http://www.ncbi.nlm.nih.gov/Traces/wgs/wgsviewer.cgi?download=GDAW01.1.fsa_nt.gz).

### Protein prediction and annotation

Transcripts were translated *in silico* from the sense strand with TransDecoder (version 2.0.1) while retaining open reading frames with hmm Pfam-A domains (release 27.0) and BLASTP alignments to (UniProt release 2015_04) [61, 63, 113]. The resulting predicted proteins were searched for previously identified protein signatures with Gene3D (version 3.5.0), PANTHER (version 9.0), Pfam (version 27.0), PIRSF (3.01), PRINTS (42.0), Prosite patterns and profiles (20.105), SMART (version 6.2), SUPERFAMILY (1.75), and TIGRFAMs (15.0) applets within InterProScan v51.0. (PRINTS, PANTHER, TIGRFAM, SUPERFAMILY, PIRSF, Gene3D, PrositeProfiles, PrositePatterns, SMART, InterProScan) [113–126]. The resulting Enzyme Commission Numbers (EC), Gene Ontology (GO) terms, and KEGG (Kyoto Encyclopedia of Genes and Genomes) pathway mapping were parsed from the InterProScan signatures. Experimental evidence based GO terms (i.e. GO evidence codes: EXP, IDA,IPO.IMP,IGI and IEP) were associated to the predicted proteins that had a BLASTP alignment to the NCBI nr (downloaded 04/02/2015) and UniProtKB (version 2015_04) databases, using the NCBI blast 2.2.29 with parameters “-evalue 1.00E-05 -max_target_seqs 100 -word_size 3 -gapopen 11 -gapextend 1 -matrix BLOSUM62 and the additional criteria of 70% percent identity and at least 50% query coverage. Taxonomic IDs of protein blast hits were counted by species. All GO term annotations were subject to GO consortium quality control taxon checks; invalid GO term associations were removed following GO consortium recommendations [127]. Signal peptides and transmembrane helixes were identified with SignalP 4.1 and TMHMM, respectively [128, 129]. GO term enrichment for the potential secreted proteins i.e. proteins that contain a predicted signal peptide and lack transmembrane helices, was conducted using GOStats [130].

### Comparative analysis

Genomes and predicted proteins were downloaded for *D*.*melanogaster* (Diptera), the confused flour beetle *T*. *castaneum* (Coleoptera), and the pea aphid *A*. *pisum* (Hemiptera) from the NCBI Genomes database. Additionally, the WTPB TSA assembly (version GBHO00000000.1) and the available 31,830 protein sequences in Uniprot-KB (accessed 5/13/15) were obtained for analysis. Completeness of the TPB and WTBP transcriptomes as well as the reference genomes from the genome projects (above) were assessed using Core Eukaryotic Genes Mapping Approach (CEGMA) version 2.5 [131]. TPB predicted proteins were aligned with BLASTP to the predicted proteomes and WTPB protein sequences. Only alignments with a bitscore of ≥100 were considered further.

### Proteins of interest

Proteins of interest involved in functions such as digestion, detoxification, inhibition of plant defense, digestion of cell wall components and sugar metabolism (previously described by Cooper et al., 2013 [51] and Stafford-Banks et al., 2014 [48]) were aligned with BLASTP to the TPB_SG1 predicted proteins only alignments with a bitscore ≥ 60 were accepted [48, 51].

### Polygalacturonases

Full length polygalacturonase protein sequences previously reported for mirid bugs *A*. *lucorum*, WTPB, and TPB as well as 17 full-length protein polygalacturonase TPB_SG1 sequences were subjected to a phylogenetic analysis using procedures described by Zhang et al. (2015) for the characterization of polygalacturonase genes in *A*. *lucorum* [50]. The presence of the polygalacturonase domain was confirmed in all the Mirid bug polygalacturonase sequences with the NCBI Conserved Domain (CD) search of the NCBI Conserved Domain Database (CDD) v3.13, default parameters [132]. The *Macrophomina phaseolina* MS6 glycoside hydrolase protein sequence EKG21667.1 served as the outgroup [50]. MEGA 6.0 software was used to align the complete polygalacturonase protein sequences with ClustalW and construct a neighbor-joining phylogenetic tree, using default parameters, with the single exception of bootstrapping the tree with 1000 replicates [133–135]. The phylogenetic tree was visualized with MEGA 6.0 software [135].

## Supporting Information

S1 Fig Agarose gel (A) and Agilent Bioanalyzer (B) images of extracted RNA used in the study. (TIF)

S1 Table *Lygus lineolaris* Blast alignments to *Acyrthosiphon pisum*, *Drosophila melanogaster*, *Tribolium castaneum*, and *Lygus hesperus*. (XLSX)

S2 Table *Lygus lineolaris* salivary gland transcriptome Gene ontology (GO) terms annotations. (XLSX)

S3 Table *Lygus lineolaris* salivary gland transcriptome Gene Ontology (GO) Molecular Function term enrichment for secreted proteins. (XLSX)

S4 Table *Lygus lineolaris* salivary gland transcriptome KEGG pathway and enzyme annotations. (XLSX)

S5 Table *Lygus lineolaris* salivary gland transcriptome InterProScan Pfam annotations. (XLSX)

S6 Table *Lygus lineolaris* salivary gland transcriptome predicted proteins of interest Blast alignment results. (XLSX)
