# Supplementary figures and images for: Insight into the Salivary Gland Transcriptome of Lygus lineolaris (Palisot de Beauvois)
Source: PLoS One. 2016 Jan 20;11(1):e0147197. doi: 10.1371/journal.pone.0147197 (PMC4720363; doi:10.1371/journal.pone.0147197)

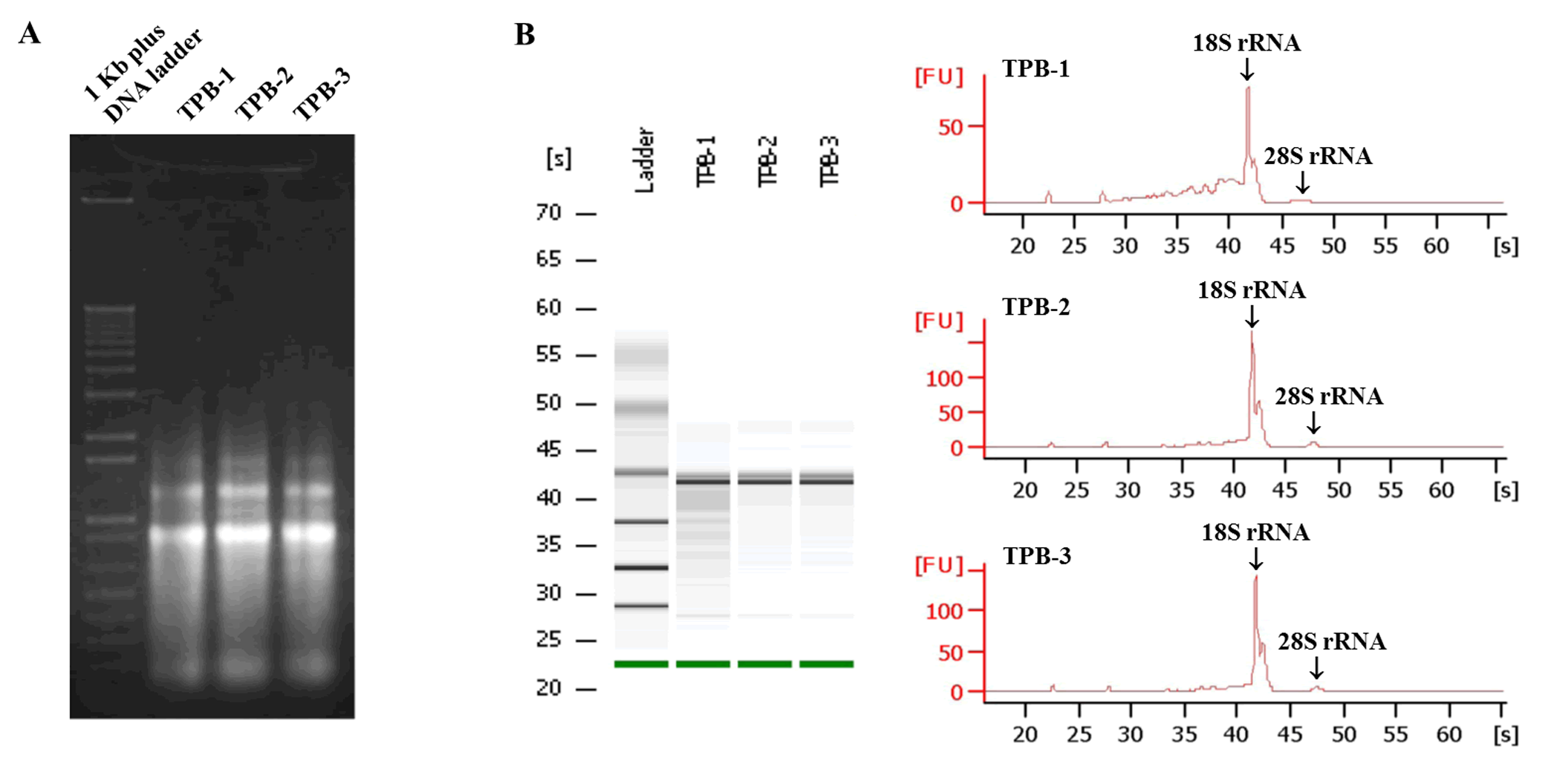

Supplement: S1 Fig — (TIF) [file pone.0147197.s001.tif]
